# Usability: An introduction to and literature review of usability testing for educational resources in radiation oncology

**DOI:** 10.1016/j.tipsro.2022.09.001

**Published:** 2022-09-17

**Authors:** Heather L. Keenan, Simon L. Duke, Heather J. Wharrad, Gillian A. Doody, Rakesh S. Patel

**Affiliations:** a School of Clinical Medicine, University of Cambridge, United Kingdom; b Education Centre, University of Nottingham, United Kingdom

**Keywords:** Education, Radiotherapy, Usability, Online, Learning, Patient

## Abstract

Usability, or the ease with which something can be used, is a key aspect in ensuring end-users can achieve the best possible outcomes from a given educational resource. Ideally usability testing should take place iteratively throughout the design of the resource, and there are several approaches for undertaking usability testing described in the wider literature. Within radiation oncology education, the extent to which usability testing occurs remains unclear. This literature review aimed to assess current practice and provide a practical introduction to usability testing for educational resource design within radiation oncology.

Two web databases were searched for articles describing planned or completed usability testing during the design of a radiation oncology educational resource. Fifteen studies were identified. Data was gathered describing the type of usability testing performed, the number of cycles of testing and the number of test subjects. Articles described design of educational resources for both patients and trainees, with the number of test subjects ranging from 8 to 18. Various testing methods were used, including questionnaires, think aloud studies and heuristic evaluation. Usability testing comprised a range of single cycle through to several rounds of testing.

Through illustrative examples identified in the literature review, we demonstrate that usability testing is feasible and beneficial for educational resources varying in size and context. In doing so we hope to encourage radiation oncologists to incorporate usability testing into future educational resource design.

## Introduction

The past two decades have seen a meteoric rise in the use of digital methods and resources within medical education [1], an increase which has been further fuelled by the Covid-19 pandemic and the accompanying restrictions on face-to-face teaching [2], [3], [4]. In parallel, digital educational resources have become an important source of accurate and up-to-date information for patients [5], [6].

Usability testing focusses on ensuring the user of any digital tool or information system can navigate and engage with the resource easily and effectively. It is widely performed in system design within the software industry and its importance in design of educational interventions is increasingly recognised [7]. It is not clear to what extent usability is applied within radiation oncology.

The aims of this article are to outline the main ways of testing usability and assess how this has been done already within radiation oncology by means of a literature review. In doing so we aim to provide a practical guide for readers of this special issue in education to incorporate usability testing into design of their own radiation oncology educational resources in future.

## Background to Usability

### What is Usability?

In its simplest terms, usability is *“the ease with which a person can use a product in a particular set of circumstances”* [8]. Usability has been more formally defined by the International Organization for Standardization as *“The extent to which a product can be used by specified users to achieve specified goals with effectiveness, efficiency, and satisfaction in a specified context of use”* [9].

In her introductory book on the subject, Barnum highlights the importance of these specified users, specified goals and specified context when considering usability [10]. To consider these within the realm of medical education:

• Users – An online oncology educational resource may be highly usable for a young adult, but relatively unusable to an elderly person (statistically more likely to be diagnosed with cancer and therefore receive radiotherapy) who does not regularly access the internet [11].
• Goals – An educational resource designed to facilitate oncology treatment decisions for patients, which contains detailed information but provides no final summary page, may be highly informative but ultimately unusable for its defined goal as users struggle to remember and assimilate what they have read.
• Context – A resource designed to be used on a Safari or Chrome web-browser but released in a hospital where all computers operate a legacy version of Internet Explorer, may be useless in its specified context.

Within this review, we will be considering the assessment of usability of educational resources within radiation oncology. Users are therefore usually either patients or healthcare practitioners and goals range from facilitating treatment decisions to improving communication or radiotherapy contouring skills.

### Why is Usability Important?

Usability is relevant in the design of any educational resource, but its importance is never clearer than within e-learning where users may be required to interact with complex systems. These systems can provide new educational opportunities, for example educational contouring software which can provide direct user feedback for trainees [12]. As complexity increases however, so too does the opportunity to lose users due to issues with system design.

Usability has been shown to be a key factor (alongside perceived usefulness) influencing our acceptance of information technology [13], [14], which predicts actual use.

E-learning has been extensively studied within the corporate field where e-learning courses increasingly replace traditional instructor-led courses [15]. While e-learning courses are often cheaper and more convenient, they have also been shown to have higher attrition rates than traditional courses; one reason for this could be poor usability of the resources [16]. Sandars agrees with this view, when he argues that poor usability could be an explanation for the findings of a 2008 meta-analysis that demonstrates that while e-learning in healthcare is superior to no intervention, it is no more effective than traditional learning methods [8], [17].

It is easy to assume that an educational resource we have created is usable; we have, after all, been carefully developing it for weeks or months and its intricacies are second nature. Barnum observes that *“From the moment you know enough to talk about a product […] you know too much to be able to tell if the product would be usable for a person who doesn’t know what you know.”* [10] It is therefore essential that we not only consider usability during the design of an educational resource, but that we formally test it.

### How to Test Usability

Sandars describes four main dimensions that we should consider when assessing usability of e-resources: the learner, technological aspects, instructional design aspects, the context [8]. It is essential that, at least in some stages, a usability assessment involves the intended end-user and that ideally it is assessed in the context in which it will finally be used. ‘Technological aspects’ refers to factors such as the ease of navigation, consistency of layout and clarity of the visual design. Instructional design includes the content itself, the interactivity and judicious use of multimedia.

There are multiple different methods of usability testing described in the literature. Table 1 summarises those most encountered within medical education, briefly describes the benefits and limitations of each and provides an example study which can be consulted for further reference.

**Table 1:** Summary of different methods of evaluating usability.

Evaluation Method	Description	Benefits (+)/Limitations (–)	Example Study
*Direct observation – live or recorded evaluation*			
Heuristic evaluation [19]	Usability experts examine an interface against a set of pre-defined characteristics - “heuristics” – such as simple language, consistency and shortcuts in order to identify usability flaws and severity	+ Quick and cheap to do in contexts where a usability expert is available + Standardised assessment method – Can identify problems which do not trouble the end-user – Requires usability expert	Randomised controlled trial of online education modules to facilitate effective family caregiver involvement in oncology [21]
Cognitive walkthrough	Experts simulate new users by carrying out typical tasks in an interface in a logical manner	+ Effective in identifying severe problems – Strict structure of tasks does not allow for exploration – Requires usability expert, ideally with background in cognitive psychology	Web-based comprehensive head and neck cancer patient education and support needs program [22]
Semi-structured interview and focus groups	Users are given the opportunity to navigate a resource then asked about its content, layout, ease of use etc. Thematic analysis may be performed.	+ Involves end users – Relies on users’ opinions rather than observed behaviour – Transcription and thematic analysis is time-consuming	Online fertility preservation decision aid for female cancer patients [23]
Think aloud	Users are asked to perform a representative task and encouraged to speak their thoughts out loud as they do so. Steps and thoughts are recorded and subsequently analysed.	+ Involves end users and directly observes their behaviour – Time intensive, particularly if formal thematic analysis is carried out – Can miss issues not directly related to task being performed	Collaborative re-design of a hospital website [24]

*Questionnaires (selected)*			
System usability scale (SUS) [25]	Well-validated ten item questionnaire where users rate statements on a five-point scale	+ Easy to use, cost-effective, can quickly survey a large cohort + Freely available – Relies on user perception – Provides non-specific information on usability issues	Testing the utility of an interactive 3D contouring atlas [26]
Usability Metric for User Experience (UMUX)-LITE [27]	Two item questionnaire which correlates well with SUS, designed to be incorporated into a larger questionnaire		Randomised controlled trial of online education modules to facilitate effective family caregiver involvement in oncology [21]
Unified Theory of Acceptance and Use of Technology (UTAUT) [28]	Technology acceptance model which identifies four key constructs which impact usability and develops statements to test these		Development and validation of a patient decision aid for prostate cancer therapy [29]

It is relatively straightforward to survey many individuals with a questionnaire; it is considerably more resource intensive to perform multiple think aloud studies, cognitive walkthroughs or heuristic evaluations. Nielson and Landauer analysed 11 usability studies using either heuristic evaluators (i.e. usability experts) or end-user evaluators (e.g. patients or clinicians), compared the number of evaluators with the number of usability problems identified, and developed a model to determine how many testers were required [18]. They showed that for a small project the optimum cost-benefit analysis requires only four evaluators. Five evaluators are generally accepted to be able to identify ∼85% of usability issues [19]. Later authors have however stressed the importance of context and appropriate sampling in defining the numbers to be studied [20].

## Literature review of usability in radiation oncology education

### Methods

**Fig. 1:**
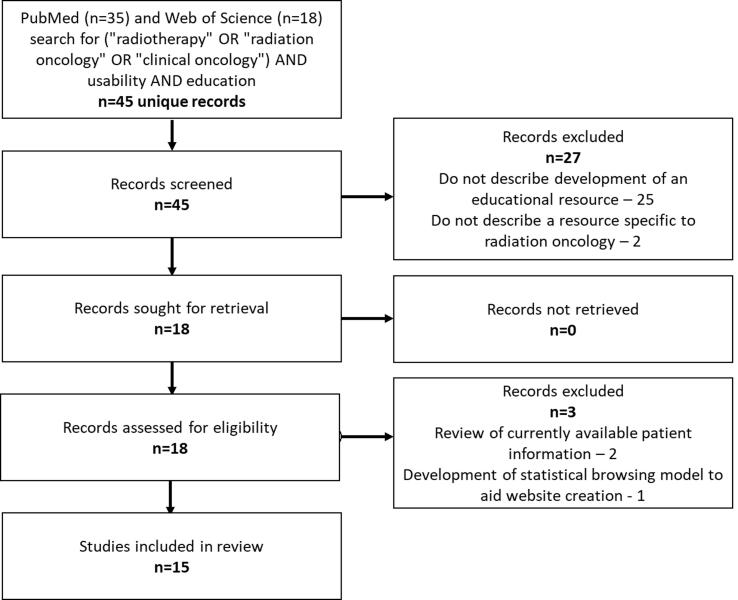
Literature review methodology for identification of relevant articles.

A literature review was carried out to assess the reported use of usability testing in the radiation oncology education literature. PRISMA guidelines were followed [30]. Inclusion criteria were articles which described completed or planned usability assessment of an educational resource within radiation oncology. The search was initially carried out on 25/6/2022 and all results up to this date were included. Fig. 1 outlines the methodology:

### Results

Three articles were identified as relevant from abstract screening and subsequently excluded at review. One was an analysis of pre-existing web resources for patients with prostate cancer [31]. While the tool they used to assess these websites had been previously tested for usability, the websites themselves were not explicitly assessed. A second article addresses usability specifically in the context of patients with lower health literacy levels [32]. They provide recommendations for enhancing usability in a practical sense, e.g. by providing audio material alongside visual, but do not cover how to carry out usability testing. A third study observed browsing patterns of visitors seeking radiology-related information on a hospital website to develop a model which could then by applied elsewhere to improve browsing experience [33]. This was excluded as it is not specifically about the development of an educational resource.

The fifteen items selected for full review were read by two reviewers (HLK, SLD) to identify the educational tool being developed, the intended audience, the usability assessment method used, the number of testers and the point in the design process at which usability was assessed. The results are summarised in Table 2.

**Table 2:** Systematic review of current use of usability testing in the radiation oncology education literature.

Paper	Educational tool	Audience	Usability testing method	Number of participants in usability testing	Timing of testing
Tran et al. [34]	Multi-language online patient education modules in radiation therapy	Cancer patients facing language barriers	Think aloud Semi-structured interview Non-standard questionnaire	8 patients at the start of treatment	After completion of design
Buzaglo et al. [35]	Educational booklet about transitioning from active cancer treatment to monitoring	Patients on completion of active cancer treatment	Non-standard questionnaire	340 adults cancer patients finishing radical chemotherapy	After completion of design
Hopmans et al. [36]	Patient information website on stereotactic ablative radiotherapy (SABR)	Lung cancer patients referred for SABR and their relatives	Think aloud	18 then 9 patients and relatives	2 cycles during design
Deraniyagala et al. [37]	eLearning programme for contouring nodal stations of head and neck	Radiation oncology residents	SUMI questionnaire*	25 residents	After completion of design
Gillespie et al. [26]	Interactive 3D contouring atlas	Radiation oncology residents	SUS questionnaire	24 residents	After completion of design
Ankolekar et al. [29]	Patient decision aid for prostate cancer therapy	Patients newly diagnosed with prostate cancer	Questionnaire based on UTAUT Expert heuristic evaluation Think aloud SUS questionnaire	22 clinicians 19 patients 11 healthy volunteers 4 usability experts	Throughout design process
Nguyen et al. [24]	Redesign of existing hospital website	Older patients with colorectal cancer	Think aloud	10, 11 patients in two separate rounds of testing	2 cycles during design
Shinn et al. [38]	Interactive website with adherence and coping program to prevent dysphagia after radiation	Head and neck cancer patients post-radiotherapy	Not performed	Not performed	Mentioned as possible ‘future work’
Arya et al. [39]	Graphic narrative patient education tool about radiotherapy	Patients undergoing radiotherapy, particularly those with poor literacy skills	Modified SUS questionnaire	34 patients and 15 practicing oncologists	After completion of design
Berg et al. [23]	Tailored online female fertility preservation decision aid	Pre-menopausal female cancer patients	Think aloud Semi-structured interviewQuestionnaire (type not specified)	17, 10 and 21 in 3 rounds of testing, including cancer survivors, patient advocates and professionals	3 cycles of iterative testing
Bigelow et al. [40]	Web-based, patient-centred decision aid for oropharyngeal cancer treatment	Patients with oropharyngeal cancer	Non-standard questionnaire	26 – 16 physicians, 4 patient education experts, 6 oropharyngeal SCC survivors	2 cycles during design
Cruz et al. [41]	Mobile app providing information and allowing reporting of treatment side effects	Patients with breast cancer undergoing radiotherapy	Focus group Non-standard questionnaire	8 professionals including nurses, physician, medical physicists, and communication networks engineer	Single cycle during design
Juraskova et al. [21]	Educational modules on communicating with caregivers during cancer treatment	Clinicians Patients and caregivers	Expert heuristic evaluation Think aloud Future plans for UMUX-LITE	1 expert 5 clinicians 3 patient-caregiver pairings 30 clinicians, 270 patient-caregiver pairs	Throughout design process
Jabbour et al. [22]	A web-based comprehensive head and neck cancer patient education and support needs program	Patients with head and neck cancer	Cognitive walkthrough Think aloud	18 patients treated for head and neck cancer	After completion of design
Raith et al. [42]	Two different augmented reality prototypes, one for patients prior to starting radiotherapy and one for radiographers to teach patient positioning	Patients undergoing radiotherapy Radiographers	Expert heuristic evaluation Semi-structured interview	3 experts	Single cycle during design

* SUMI– Software Usability Measurement Inventory [43].

Most examples of usability testing in the radiation oncology medical education literature describe the design of a patient information resource (13/15, 87%), although there are also examples of usability assessment use in designing resources for healthcare professionals (4/15, 27%). There are two examples of papers which do both: Juraskova et al. [21] describe the design of educational modules for both clinicians and patients/caregivers, and the study by Raith et al. [42] describes two separate augmented reality protocols for patients and radiographers respectively.

Direct observation was used in 8/15 (60%) studies. 3/15 (20%) describe the use of expert heuristic evaluation. Two of these studies are among those which describe more extensive usability testing, including multiple testing modalities and iterative testing throughout the design process. 6/15 (40%) studies describe a think aloud. These involve a range of 8 to 18 participants in any single round of testing, and some studies describe more than one round. The formality of analysis of the think aloud varies; some papers describe extensive transcription and thematic analysis, whereas others describe drawing general learning points.

10/15 (67%) studies use a survey as part of their usability assessment. Of these, 5/10 (50%) were not formally validated usability questionnaires, but asked questions explicitly designed to probe usability. Of those which used validated studies, three (60%) used the SUS (one of which was modified) and one (20%) used SUMI. One study mentions a future plan to use UMUX-LITE but has not yet done so.

6/15 (40%) studies described more than one round of usability testing during the design process and 7/15 (47%) studies use more than one type of usability assessment. Three studies – Juraskova et al., [21], Ankolekar et al., [29] and Berg et al., [23] – did both.

Although most studies assessed usability on the intended target audience this was not universal; there was one example of a study where clinicians alone were used to assess the usability of a resource being designed for patients. Raith et al., [42] were unable to assess their augmented reality resource on patients due to the restraints of the covid-19 pandemic.

## Discussion

In the introduction to this article, we described five methods of usability testing. These can be divided into ‘expert-led’ testing (heuristic evaluation, cognitive walkthrough) and ‘user-led’ testing (think aloud, semi-structured interview, questionnaire). Examples of all five types of testing exist in the current radiation oncology education literature. There are notably far more cases of user-led testing.

Usability experts may come with a cost - much of the seminal work on usability comes out of software design where usability experts to undertake heuristic evaluations are readily available, which is likely to be a luxury unavailable in healthcare. This scarcity may be addressed by collaboration with computer science departments within or across academic institutions.

In one of only three studies employing expert-led testing, Ankolekar et al. describe the development and validation of a patient-led decision aid for prostate cancer [29]. They detail an extensive process of usability testing involving multiple cycles of testing, re-design and re-testing. Over five rounds of testing, they employ questionnaires, heuristic evaluation and think-aloud methods. They explain that *“Our development process spanned over two years and involved 58 participants, resulting in >100 h of interview material and feedback that needed to be processed, analyzed and incorporated in successive rounds.”* While such extensive testing clearly has the potential to produce a high-quality educational resource, the time and financial commitment required may prove a disincentive for others to carry out similar work.

Other studies describe less extensive usability testing from which the authors are nevertheless able to make changes to their resource. Bigelow et al. describe two cycles of questionnaire-based testing and demonstrate an improvement in usability between cycle 1 and cycle 2 following changes to the design, language and graphics of their resource [40]. We would therefore argue that current literature suggests that it is both valuable and feasible to carry out usability studies within radiation oncology education on a variety of different scales suited to the specific aims of the resource.

Most of the studies which carry out only one or two rounds of usability testing employ user-led testing. Many of the advantages of this are intuitive; testing on the group that will ultimately be using the resource makes logical sense. Additionally, most healthcare professionals involved in the design of an educational resource will have easy and free (limited by necessary ethical approval) access to the end users, be they trainees or patients.

Within user-based usability testing a scale of resource and time-intensity exists. Formal think-aloud testing is an extensive process involving scriptwriting, taping of users, transcription of tapes, coding by independent coders and thematic analysis. Hopmans et al. provide an example of how this process can be shortened while still providing valuable insights [36]. In an initial round of think-aloud testing they ask 18 participants to navigate through their website and then ask a series of probing questions. They transcribe all the interviews, then three are selected for independent coding and analysis by two separate researchers; the remainder are analysed by one researcher only. This is one example of how a potentially intensive analysis process can be shortened while still providing useful insights. The article does not describe how the three transcriptions are selected; it would be important to ensure this is done at random.

Finally, questionnaires are a relatively easy method of usability testing. Most frequently used in our review is the SUS, which is a well-validated usability survey that is freely available online. As healthcare practitioners we are generally used to seeking survey style feedback on our educational resources, so it is fairly straightforward to add in some usability-focussed questions. If longer surveys like UTAUT are felt to be too arduous and add excessively to survey burden, then the SUS or UMUX-LITE are shorter alternatives.

Several studies in our review test usability on ‘cancer patients’. We would agree that it is essential in health education to test resources on the target audience, however it is worth considering that unless these testers are at the beginning of their cancer journey, they may in fact have a higher level of knowledge than intended. In this context, healthy volunteers may provide a reasonable alternative, with the caveat that volunteers are likely to be interested and engaged in healthcare and may therefore have a higher level of health literacy than the general population.

Our systematic review identified only two studies describing usability testing on an educational resource for radiation oncology trainees. This might simply represent the fact that there are fewer such resources being regularly created. The two articles identified in our review [26], [37] both describe design of resources to help with contouring. This is clearly a field where usability is of crucial importance as factors like ease of navigation, ability to concurrently view atlases and contouring software and similarity to trainees’ local contouring software is likely to have a large impact on their engagement with the resource. We would encourage anyone designing such a resource to consider undertaking and reporting usability testing.

As a result of the inclusion criteria, all articles included in our review include a description of planned or completed usability testing. Only a proportion of these articles describe whether and how the results of the usability testing benefited ongoing resource development. An example of this being done well is Nguyen et al.; Table 1 in their paper describes the method, results and insights of serial rounds of usability testing [24]. We would suggest that a description of the changes made (and, if possible, repeat usability testing to demonstrate an improvement), would enhance any paper reporting usability as it would help identify common areas of difficulty.

### Limitations and possible future work

A limitation of this review is that due to the search criteria, it only picks up studies which have specifically mentioned ‘usability’. It is possible that usability may be assessed but not formally described, or that usability is being assessed in educational resources that are never formally published (or are published only in abstract form). We would encourage more radiotherapy researchers to publish their usability data and lessons, as these may help prevent others from repeating similar mistakes. The literature search was limited to only two databases, which may also have limited the number of results.

A future review might identify a more specific area of radiation oncology educational material and assess all educational resources published within this field, to determine what proportion of them report usability testing. This would give a better idea of how widespread usability testing is. This is not possible to assess from our review, which does not include educational resources which are not usability tested.

## Conclusion

In this article, we have discussed the rationale for carrying out usability testing in the design of educational resources and described the main methods for doing so. We go on to report the results of a literature review of the current use of usability testing within radiation oncology.

Current practice demonstrates that there is a balance to be achieved between the resource intensity of usability testing and the potential improvements to an educational resource. We would encourage all educationalists designing resources for either patients or trainees to consider how usability testing might reasonably be incorporated in their own design process. The ideal method(s) depends on the aim of the resource and certainly anyone aiming to design a durable and far-reaching resource should consider multiple rounds and methodologies of usability testing.

We hope we have also provided the necessary tools and information to show that even in simpler more local projects, it is feasible to carry out some basic usability testing to maximise the impact of a resource.

## Declaration of Competing Interest

The authors declare that they have no known competing financial interests or personal relationships that could have appeared to influence the work reported in this paper.
